# Prognosis and tumor grading in bladder urothelial carcinomas with intratumoral grade heterogeneity

**DOI:** 10.3389/pore.2026.1612553

**Published:** 2026-10-05

**Authors:** Gökhan Veli Alkan, Kutsal Doğan, Meltem Türk Alkan, Evrim Önder

**Affiliations:** 1 Department of Pathology, Faculty of Medicine, Ordu University, Ordu, Türkiye; 2 Department of Pathology, Faculty of Medicine, Hacettepe University, Ankara, Türkiye; 3 Department of Pathology, Ankara Etlik City Hospital, Ankara, Türkiye

**Keywords:** bladder cancer, grading, heterogeneity, invasion, uro-oncology

## Abstract

**Aims:**

Grade heterogeneity is one of the most debated issues in the grading of urothelial carcinomas. The 2022 WHO classification states that approximately one-third of urothelial carcinomas exhibit grade heterogeneity and suggests that the presence of ≥5% high-grade component may justify classification as high-grade. Nevertheless, the need for further evidence was also emphasized. This study aimed to evaluate the prognostic impact of the high-grade component in urothelial carcinomas with grade heterogeneity and to determine a threshold value for high-grade proportion that is clinically meaningful for grading.

**Methods:**

This retrospective study included 180 cases of non-muscle-invasive urothelial carcinoma with grade heterogeneity, diagnosed between January 2019 and August 2022 on TUR specimens, with available clinical data. Recurrence and survival time were assessed. Cases with >90% high-grade component were excluded.

**Results:**

Recurrence was observed in 26.7% of cases. The two-year survival rate was 73.3%. While no significant association was found between recurrence and the proportion of high-grade component, survival time significantly decreased as the proportion of high-grade component increased. ROC curve analysis identified 27.5% as the cut-off value associated with adverse prognosis.

**Conclusion:**

In non-muscle-invasive urothelial carcinomas, the proportion of high-grade component showed a strong association with survival. Together with literature findings, our results suggest that a threshold higher than 5% may be more appropriate for assigning tumors to the high-grade category. Nonetheless, risk stratification incorporating tumor size and extent remains essential for prognostic evaluation.

## Highlights

- Grade heterogeneity is observed in a substantial proportion of urothelial carcinomas and remains a major source of diagnostic variability.
- Higher proportions of high-grade component were associated with shorter survival, with 27.5% identified as a potential prognostic threshold.
- A threshold higher than 5% for defining high-grade tumors may improve grading consistency and prognostic accuracy in clinical practice.

## Introduction

Histological grading of urothelial carcinomas (UC) is one of the key determinants in guiding treatment protocols. The two-tier grading system was first introduced in the 1998 WHO/ISUP consensus report and was maintained in the 2004 and 2016 WHO classifications [1]. Most recently, the fifth edition published in 2022 has continued to adopt this two-tier grading system for UC. Studies comparing low- and high-grade UC have shown that this system also correlates with the prevalence of genetic alterations. However, in light of accumulating molecular evidence, some reports suggest that the three-tier grading system, originally used in the 1973 classification, demonstrates a stronger correlation with both clinical outcomes and genetic alterations [2]. In fact, based on these findings, it has recently been proposed that the three-tier classification may be more appropriate for clinical use [3]. Building on these observations, a hybrid four-tier system that subdivides the high-grade category into G2 (Grade 2) and G3 (Grade 3) has also been proposed [4]. One of the main arguments for these proposals is that the three-tier system provides higher interobserver reproducibility compared to the two-tier system.

Among the most debated aspects of histological grading in UC is grade heterogeneity [5]. Various studies have reported that 5%–30% of non-invasive carcinomas contain both low-grade and high- grade components [6, 7]. In such cases, deciding which component should determine the final tumor grade is of critical importance. Grade heterogeneity further reduces interobserver reproducibility, a problem compounded by the inherent subjectivity of the histological parameters used to distinguish low-grade from high-grade lesions [8].

Although the histological grading system of UC has remained unchanged since the 2004 WHO classification, the prognostic relevance of grade heterogeneity has become an increasingly debated issue in recent years [9]. According to the 2004 WHO classification, non-invasive urothelial carcinomas with grade heterogeneity were classified in the high-grade category. However, some studies have shown that the prognosis of such tumors may resemble that of low-grade carcinomas rather than high-grade carcinomas [6].

The 2022 WHO classification of urinary and male genital tumours specifically highlights intratumoral grade heterogeneity as a problematic area for pathologists, noting that it often results in a higher frequency of high-grade diagnoses [10]. While molecular studies may eventually provide more definitive insights, it is currently recommended to adopt a standardized approach by considering a defined cut-off proportion of high-grade component [11]. The 2022 WHO classification states that approximately one-third of UC display grade heterogeneity and suggests that if a low-grade tumor contains ≥5% high-grade component, the tumor should be graded as high-grade. Conversely, tumors with <5% high-grade component should be reported as “low-grade UC with <5% high-grade component.” In addition, all histological subtypes of UC and those with divergent differentiation are classified in the high-grade category [12].

In this study, we aimed to investigate the prognostic impact of the high-grade component in UC with intratumoral grade heterogeneity and, based on prognostic data, to determine a threshold value for the high-grade proportion with meaningful implications for grading.

## Materials and methods

Transurethral resection of bladder tumor (TURBT) specimens diagnosed between January 2019 and August 2022 were retrieved from the archives of the Ministry of Health Ankara Dışkapı Training and Research Hospital. All hematoxylin and eosin slides were re-evaluated according to the 2022 WHO Classification of Urinary System Tumors. The proportion of low- and high-grade components and the presence of lamina propria invasion were recorded. Cases with >90% high-grade component, without any high-grade component, muscle-invasive tumors, carcinoma *in situ*, specific histological subtypes, and recurrent carcinomas initially diagnosed before 2019 were excluded.

In total, 184 cases were identified. Survival, recurrence, and progression were evaluated. Progression was defined as an increase of ≥5% in the high-grade component or the presence of muscle invasion in follow-up specimens (re-TUR or cystectomy). Four cases were excluded due to missing clinical data, leaving 180 for analysis. Recurrence-negative status required a minimum follow-up of 2 years.

Demographic and clinical data (age, sex, survival, recurrence) were obtained from the hospital’s electronic database. Statistical analyses were performed using SPSS version 27.0 (IBM Corp., Armonk, NY, USA). Continuous variables were summarized as mean ± standard deviation, categorical variables as frequency and percentage. Group comparisons were performed using the independent samples t-test, and ROC curve analysis was used to determine cut-off values. Statistical significance was set at p < 0.05.

## Results

The study included 180 patients, 156 (86.7%) male and 24 (13.3%) female, with a mean age of 65.9 ± 10.5 years (range 45–90). The mean high-grade component proportion was 33.7% ± 29.8% (median 22.5; range 2–80) (Figure 1). Lamina propria invasion was present in 25% of cases (n = 45). Recurrence occurred in 48 patients (26.7%), while 132 (73.3%) remained recurrence-free. The mean recurrence- free interval was 10.3 ± 5.7 months, and the mean overall survival was 14.6 ± 10.1 months. The two- year survival rate was 73.3% (Table 1).

**FIGURE 1:**
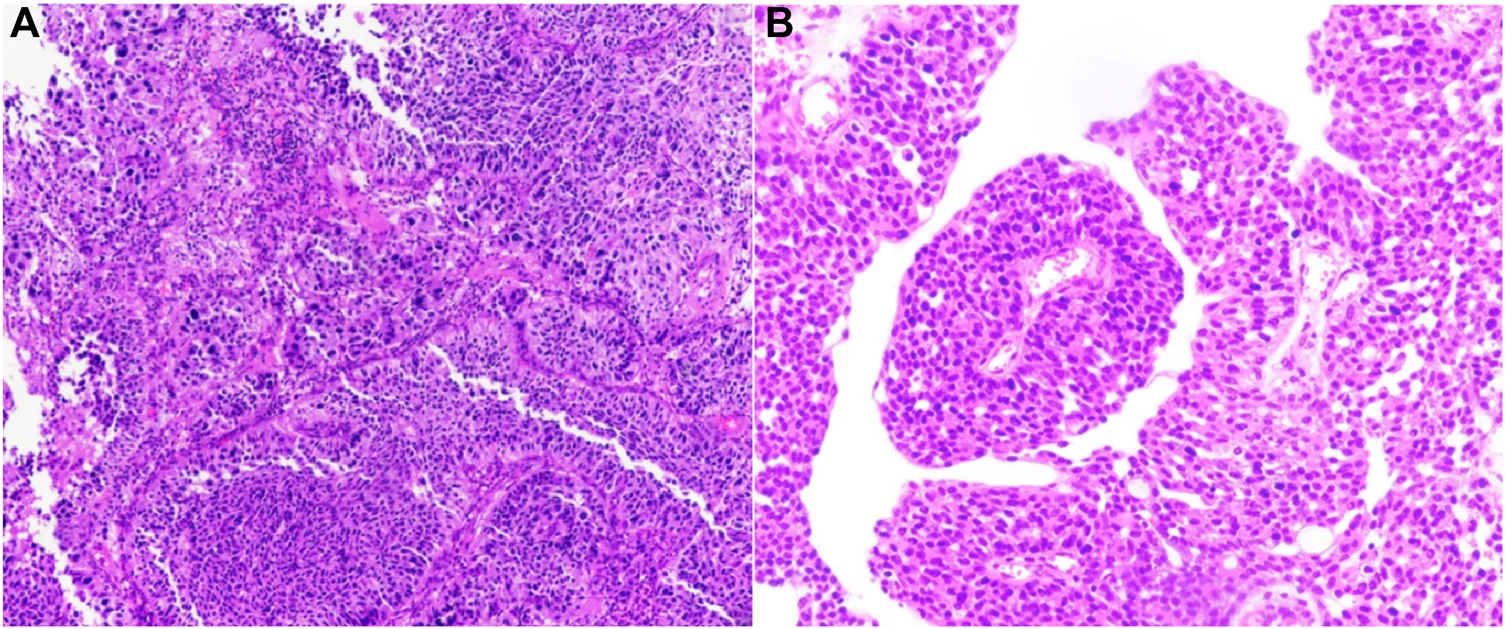
**(A)** Urothelial carcinoma with focal high-grade component (H&E ×100). **(B)** Low- grade urothelial carcinoma with a high-grade area (H&E ×200).

**TABLE 1:** Demographic, clinical, and pathological characteristics of patients with urothelial carcinoma.

Characteristics	n (%)/Median	Min-Max
Sex	Male	156 (86.7)
​	Female	24 (13.3)
Age (years)	65.9	65 (45–90)
Recurrence	Absent	132 (73.3)
​	Present	48 (26.7)
Lamina propria invasion	Absent	135 (75.0)
​	Present	45 (25.0)
High-grade proportion (%)	33.7	22.5 (2–80)
Recurrence-free interval (months)	10.3	9.5 (3–19)
Survival time (months)	14.6	15 (1–32)

Survival-based analysis revealed significant differences: patients who survived <2 years had a higher mean proportion of high-grade component (46.4% vs. 29.1%,p = 0.045) and were older (72.6 vs. 63.4 years, p = 0.002), although recurrence-free interval did not differ between these survival groups. Recurrence status, on the other hand, was not associated with significant differences in high-grade proportion, age, or survival time (p > 0.05) (Table 2).

**TABLE 2:** Comparison of demographic and clinicopathological characteristics according to recurrence status, 2-year survival, and lamina propria invasion.

Comparison	Group 1	Group 2	p-value
Recurrence	Recurrence	No recurrence	​
High-grade proportion (%)	37.6 ± 31.6	32.3 ± 29.4	0.549
Age (years)	63.3 ± 9.9	66.8 ± 10.6	0.254
Survival (months)	15.8 ± 7.7	14.1 ± 11.3	0.765
2-year survival	<2 years	≥2 years	​
High-grade proportion (%)	46.4 ± 28.4	29.1 ± 29.2	0.045
Age (years)	72.6 ± 10.4	63.4 ± 9.5	0.002
Recurrence-free time (months)	9.2 ± 5.4	10.7 ± 6.0	0.634
Lamina propria invasion	Invasion	No invasion	​
High-grade proportion (%)	51.3 ± 31.5	27.8 ± 27.1	0.007
Age (years)	74.1 ± 11.7	63.1 ± 8.5	<0.001
Recurrence-free time (months)	12.0 ± 6.2	9.9 ± 5.7	0.572
Survival (months)	15.9 ± 10.6	13.7 ± 10.2	0.681

Group 1 and Group 2 represent the categories defined separately for each comparison: recurrence vs. no recurrence, <2 years vs. ≥2 years survival, and lamina propria invasion vs. no invasion.

Lamina propria invasion was associated with a significantly higher high-grade proportion (51.3% vs. 27.8%, p = 0.007) and older age (74.1 vs. 63.1 years, p < 0.001), while recurrence-free interval and overall survival did not differ significantly between groups (Table 2).

ROC curve analysis demonstrated that a high-grade component ≥27.5% (AUC = 0.682, p = 0.032) and age ≥66.5 years (AUC = 0.756, p = 0.003) were significant predictors of shorter survival. In contrast, no significant cut-off values were identified for recurrence with respect to high-grade proportion, age, or survival time (p > 0.05). Recurrence-free interval was also not a significant predictor (p > 0.05). Kaplan–Meier survival analysis similarly showed a trend toward worse survival in patients with >27.5% high-grade component, although this was not statistically significant (log-rank p = 0.061) (Figure 2).

**FIGURE 2:**
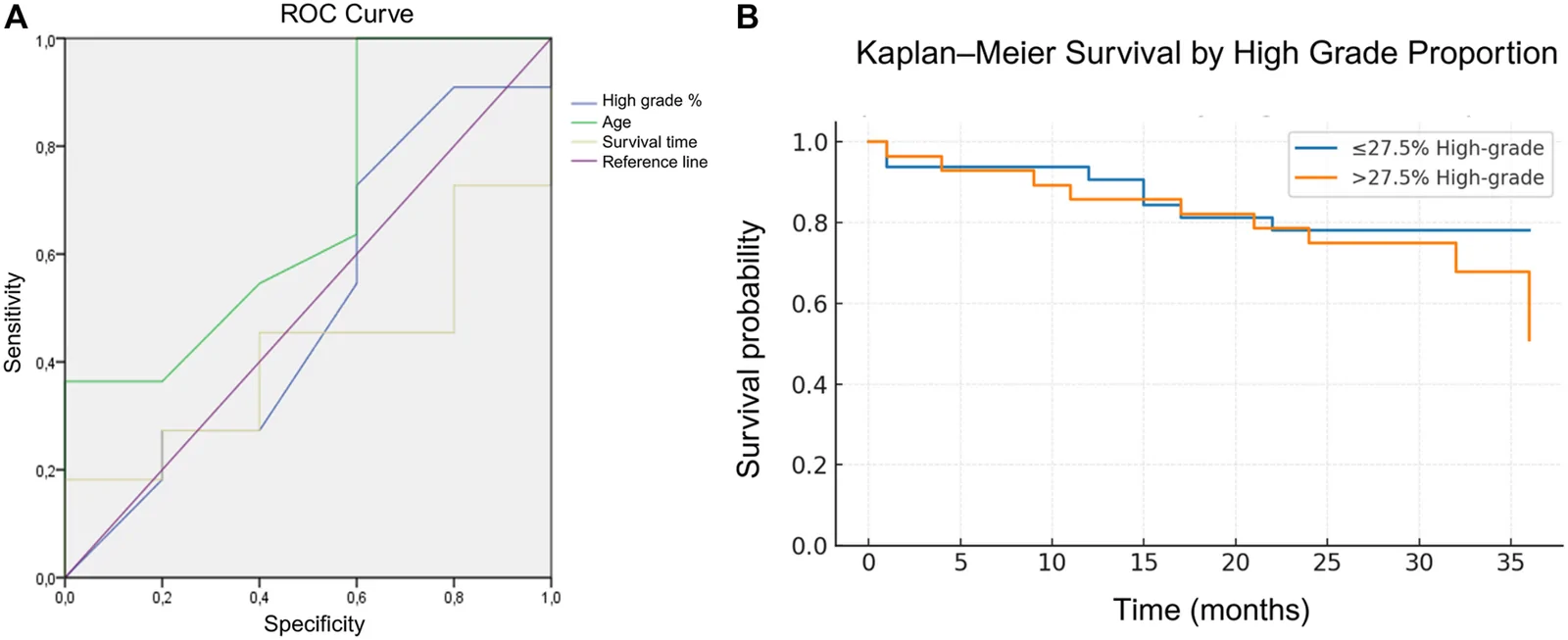
**(A)** ROC curve analysis. High-grade component ≥27.5% and age ≥66.5 years were significant predictors of shorter survival, while survival time was not predictive. **(B)** Kaplan–Meier survival analysis similarly showed a trend toward worse survival in patients with >27.5% high-grade component.

## Discussion

According to the most recent 2022 WHO classification of tumors of the urinary system and male genital organs, grade heterogeneity is observed in approximately one-third of UC [1]. It has generated considerable debate, particularly regarding the grading of low-grade UC that contain a limited proportion of high-grade component. One of the main reasons why the two-tier (WHO 2004) and three-tier (WHO 1973) systems continue to be compared is precisely this diagnostic challenge [13].

It has been reported that, when the 2004 and 1973 grading systems were compared, UC classified as grade 3 in the 1973 system showed higher rates of progression compared with tumors categorized as high-grade in the two-tier system. This difference was attributed to the inclusion of tumors with only focal high-grade components in the high-grade category under the 2004 system. It was further emphasized in the same study that the 2004 classification may result in lower reproducibility when applied to such heterogeneous tumors. It was also noted that, in some cases, degenerative changes mimicking high-grade features may further contribute to misclassification [14].

Similarly, in a multicenter study, it was shown that the 1973 classification correlated more strongly with tumor progression than the 2004 system in non–muscle-invasive bladder carcinomas (Ta/T1), although no difference was observed in recurrence rates between the two systems [15]. These observations suggest that the high-grade category in current classification represents a heterogeneous group of tumors, raising concerns that a subset of patients may receive unnecessary treatment. Determining a minimum proportion of high-grade component necessary for classification as high-grade therefore carries critical clinical importance [16].

It has been reported that tumors with grade heterogeneity tend to exhibit clinical behavior similar to low-grade tumors [6]. In another study, the response of such tumors to intravesical BCG therapy was analyzed, and higher response rates were observed compared with pure high-grade tumors, leading to the conclusion that they may represent a ‘favorable prognostic subtype’ [17]. On the other hand, it is also reported that non–muscle-invasive papillary UC with grade heterogeneity exhibited outcomes more similar to low-grade tumors, although the difference was not statistically significant [18].

The 2023 ISUP consensus report emphasized that current data support an intermediate behavior of low- grade UC with focal high-grade areas. However, consensus has not yet been reached on the exact proportion that defines “focal high-grade” 13. In a 2022 review, it was noted that multidisciplinary studies, along with emerging artificial intelligence systems, may provide clearer insights into the behavior of this group of tumors 7. The 2022 WHO classification suggests using a ≥5% threshold for designating tumors as high-grade, while at the same time emphasizing the need for further evidence [1]. In support of this approach, 160 papillary UC with grade heterogeneity were recently evaluated, and it was reported that when a 5% threshold was applied, tumors classified as high-grade demonstrated better survival compared with pure high-grade tumors but worse survival compared with pure low-grade tumors [19, 20].

In the 2021 update of the European Association of Urology (EAU) guidelines for non–muscle-invasive bladder cancer, it was emphasized that risk stratification should be applied when determining treatment procedures for this group of tumors, underscoring the importance of tumor size, multiplicity, and the presence of carcinoma *in situ* (CIS) [21].

In our study we did not demonstrate any significant corelation between high-grade proportion and recurrence. Notably, a negative correlation was observed between high-grade proportion and overall survival, and a similar negative correlation was also present between high-grade proportion and survival time.

A statistically significant association was found between lamina propria invasion and the proportion of high-grade component (p = 0.007). This finding is consistent with the expected invasive potential of high-grade tumors.

When overall survival was considered, a threshold of 27.5% for the high-grade component was calculated in our study. This threshold suggests that tumors with a high-grade component exceeding 27.5% behave more similarly to pure high-grade tumors. Kaplan–Meier survival analysis demonstrated a parallel trend, with patients harboring >27.5% high-grade component experiencing worse survival, although this did not reach statistical significance. The value identified is higher than the 5% cut-off proposed by the 2022 WHO classification; however, the need for further studies is evident. Nonetheless, in routine pathological practice, applying a higher cut-off for the high-grade component may help limit subjectivity caused by focal degenerative changes and may improve reproducibility.

Our primary aim was to evaluate the prognostic impact of the high-grade proportion and to investigate a potential threshold value; therefore, cases with tumor multifocality or coexisting CIS were not included among the 180 analyzed. In addition, the absence of tumor size data, which represents an important factor in risk stratification, can be considered a limitation of our study.

In conclusion, our findings demonstrate a strong association between the proportion of high-grade component and survival in non–muscle-invasive UC. Moreover, our study provides evidence suggesting that a threshold higher than 5% may be more appropriate when assigning tumors to the high-grade category. However, to strengthen consistency in grading and ensure appropriate treatment decisions, multicenter studies incorporating tumor size and other clinical and prognostic parameters are needed.

## Data Availability

The raw data supporting the conclusions of this article will be made available by the authors, without undue reservation.
